# Epilepsy and neurodevelopmental disorders: a review

**DOI:** 10.1192/j.eurpsy.2026.10432

**Published:** 2026-07-31

**Authors:** R. M. S. Lousada, R. Nogueira, D. Cotovio

**Affiliations:** Hospital Beatriz Angelo, Lisbon, Portugal

## Abstract

**Introduction:**

Epilepsy is characterized by recurrent seizures and encompasses a heterogeneous group of syndromes with varying etiologies. Neurodevelopmental disorders (NDDs) include autism spectrum disorder (ASD), attention-deficit hyperactivity disorder (ADHD), and intellectual disabilities (ID). Epilepsy prevalence in individuals with ID can be as high as 20%, and the incidence of ADHD in patients with epilepsy varies from 11% to 46%. Although the etiology of epilepsy is unclear in many patients, there is increasing evidence for the existence of genetic traits common to both epilepsy and neurodevelopmental disorders. These include chromosomal abnormalities, copy number variations, and single gene diseases. Understanding these overlaps is essential for accurate diagnosis, treatment planning, and prognosis.

**Objectives:**

The aim of this review is to explore the published literature addressing the shared pathophysiology and clinical implications between epilepsy and neurodevelopment disorders.

**Methods:**

A non-systematic literature review was conducted using PubMed and Google Scholar databases. The following search terms were used: “epilepsy”, “neurodevelopmental disorders”.

**Results:**

Neurodevelopmental disorders and epilepsy often coexist and share common pathological mechanisms. Syndromic conditions such as Rett syndrome, Dravet syndrome, and Tuberous Sclerosis Complex, frequently present with both epilepsy and NDDs due to underlying genetic mutations. Even in non-syndromic cases, individuals with ASD or ID have higher rates of epilepsy than the general population. Shared pathophysiological pathways involve disrupted synaptic function, altered neuronal excitability, and imbalance in inhibitory/excitatory neurotransmission. Additionally, subclinical epileptiform activity may contribute to cognitive and behavioral impairments. Epilepsy in the context of NDDs is often more resistant to treatment and associated with increased psychiatric comorbidities. Antiepileptic drugs may have variable effects on behavior and cognition, further complicating management.

**Conclusions:**

The intersection between epilepsy and neurodevelopmental disorders has significant clinical implications, particularly regarding early detection, prognostic assessment, and treatment strategies. Evidence for a biological overlap between these diseases is likely to lead to a comprehensive understanding of these disorders and the future discovery of new therapeutic targets.

**Disclosure of Interest:**

None Declared

